# Differential gene expression profiles of radioresistant oesophageal cancer cell lines established by continuous fractionated irradiation

**DOI:** 10.1038/sj.bjc.6602187

**Published:** 2004-09-14

**Authors:** K Fukuda, C Sakakura, K Miyagawa, Y Kuriu, S Kin, Y Nakase, A Hagiwara, S Mitsufuji, Y Okazaki, Y Hayashizaki, H Yamagishi

**Affiliations:** 1 Division of Digestive Surgery, Kyoto Prefectural University of Medicine, Kawaramachi-hirokoji, Kamigyo-ku, Kyoto 602-8566, Japan; 2 Division of Gastroenterology and Hepatology, Kyoto Prefectural University of Medicine, Kawaramachi-hirokoji, Kamigyo-ku, Kyoto 602-8566, Japan; 3 Genomic Sciences Center, RIKEN, Yokohama Institute, 1-7-22 Suehiro-cho, Tsurumi-district. Yokohama 230-0045, Japan

**Keywords:** radioresistance, oesophageal cancer, cDNA microarray, fractionated irradiation

## Abstract

Radiation therapy is a powerful tool for the treatment of oesophageal cancer. We established radioresistant cell lines by applying fractionated irradiation in order to identify differentially expressed genes between parent and radioresistant cells. Six oesophageal cancer cell lines (TE-2, TE-5, TE-9, TE-13, KYSE170, and KYSE180) were treated with continuous 2 Gy fractionated irradiation (total dose 60 Gy). We compared expression profiles of each parent and radioresistant lines on a cDNA microarray consisting of 21168 genes. In the fractionated irradiation trial, four radioresistant sublines (TE-2R, TE-9R, TE-13R, KYSE170R) were established successfully, and we identified 19 upregulated and 28 downregulated genes common to radioresistant sublines. Upregulated genes were associated with apotosis and inflammatory response (BIRC2 and COX-2), DNA metabolism (CD73), and cell growth (PLAU). Downregulated genes were associated with apoptosis (CASP6), cell adhesion (CDH1 and CDH3), transcription (MLL3), and cell cycle (CDK6). Some of these genes were known to be associated with radiation response, such as COX-2, but others were novel. Reverse transcription–polymerase chain reaction confirmed that genes selected by cDNA microarray were overexpressed in clinical specimens of radioresistant cases. Global gene analysis of radioresistant sublines may provide new insight into mechanisms of radioresistance and effective radiation therapy.

Radiation therapy for oesophageal cancer is effective in selected patients but there are patients who show no response and suffer from side effects such as immunosuppression. Many factors determine tumour resistance in clinical radiotherapy, including tumour size, hypoxia, and intrinsic radiosensitivity. Clinical data suggest that size is one of the most important factors to predict outcome for cancer patients after radiotherapy (Eifel *et al*, 1994). The most obvious explanation for this is related to the number of clonogenic cells that must be sterilised. However, because of heterogeneity in both patient and tumour characteristics, the volume effect is less pronounced than would be expected from a simple proportionality between the number of clonogenes and volume.

Chronic exposure of cells to an ionising radiation induces an adaptive response that results in increased tolerance to the subsequent cytotoxicity caused by this ionising radiation (Russell *et al*, 1995; Dahlberg *et al*, 1999; Pearce *et al*, 2001). Although the factor that ultimately determines a cell's radiosensitivity is quite complex, several researchers have pointed out that the induction of apoptosis is correlated with the biological effectiveness of the radiation (Dewey *et al*, 1995; Meyn *et al*, 1996). To investigate clinical radioresistance, regimens of fractionated ionising radiation (FIR) *in vitro* have been used to determine molecular mechanisms underlying radioresistance. Tumour cells are heterogeneous with respect to cure-limiting characteristics, and the radioresistant phenotype is correlated often with several factors, including alterations in cell cycle checkpoints, slowed growth, and decreased apoptosis (Coleman and Stevenson, 1996; Maity *et al*, 1997). Recently, a relationship between radioresistance and expression of several genes has been reported, including p53 (Brachman *et al*, 1993; Biard *et al*, 1994), ras (Sklar, 1988), raf-1 (Kasid *et al*, 1987), and bcl-2 (Kitada *et al*, 1996). Biard *et al* (1994) reported the first model in which cells bearing a p53 gene mutation display enhanced sensitivity to ionising radiation, although Brachman *et al* (1993) reported that the p53 gene mutation does not correlate with the radiosensitivity of 24 head and neck cancer cell lines. The activation of raf has been positively correlated with the radioresistance of head and neck squamous cell carcinomas (Riva *et al*, 1995), and certain cell cycle- and apoptosis-related genes have also been correlated with radiosensitivity; for example, loss or dysfunction of p16 renders resistance to melanoma cells against the ionising radiation, whereas the expression of exogenous wild-type p16 and p21 in glioblastoma cells can induce radiosensitivity (Hsiao *et al*, 1997; Matsumura *et al*, 1997). Overexpression of bcl-2 in multiple tumour cells increases radiation resistance (Gasparini *et al*, 1995; Weller *et al*, 1995; Mackey *et al*, 1998), and bax, the other number of the bcl-2 family, antagonises the bcl-2 function by forming a heterodimer. Although such discoveries have helped develop a partial understanding of the molecular mechanisms responsible for cellular radiosensitivity, the entire process still remains to be elucidated. The advent of microarray gene expression technology permits the simultaneous analysis of the expression levels of thousands of genes (DeRisi *et al*, 1996; Eisen *et al*, 1998; Golub *et al*, 1999). Therefore, a study on these molecular genetic events, which are related to radiosensitivity, can be conducted (Hanna *et al*, 2001; Kitahara *et al*, 2002). This may also lead to identifying and gene regulatory pathways resulting in the development of cell resistance to therapeutic procedure. However, little is known about the clinical significance of these genes in order to estimate radiotherapy effectiveness.

We have tried to identify differentially expressed genes between radiosensitive and radioresistant oesophageal cancer cell lines. Radioresistant sublines were established by FIR, and we applied cDNA microarrays to the parent and radioresistant sublines. Finally, for the clinical application of selected genes, we studied the expression status of COX-2 and BIRC2 mRNA in biopsy specimens obtained from ‘radio-effective’ and ‘noneffective’ cases of oesophageal cancer.

## MATERIALS AND METHODS

### Cells and cell culture

The human oesophageal squamous cancer cell lines TE-2, TE-5, TE-9, and TE-13 were obtained from the Cell Resource Center for Biomedical Research Institute of Development, Aging and Cancer (Tohoku University, Sendai, Japan) (Nishihira *et al*, 1993). Cell lines KYSE170 and KYSE180 were kindly provided by Dr Shimada (Kyoto University, Kyoto, Japan) (Shimada *et al*, 1992). Cells were cultured in RPMI-1640 (Life Technologies, Grand Island, NY, USA) with 100 U ml^−1^ of penicillin. 100 *μ*g ml^−1^ of streptomycin, and 10% fetal bovine serum, and incubated at 37°C in 5% CO_2_/95% air.

### Establishment of radioresistant cell lines

The cells were first grown to approximately 50% confluence in vented 25-cm^2^ culture flasks. Cells were treated with 2 Gy of X-ray irradiation (120 kV and 3.5 mA for 4 min with a 0.5-mm A1 filter) using an X-ray generator (M-150 WE: SOFTEX Co., Tokyo, Japan) and then returned to the incubator. When they reached approximately 90% confluence, the cells were trypsinised and subcultured into new flasks. When they reached approximately 50% confluence, the cells were again irradiated (second fraction). The fractionated irradiations were continued until the total doses reached 60 Gy. The parental cells were subjected to identical trypsinisation, replating, and culture conditions but were not irradiated. For all assays on irradiated cells, there was at least a 2-week interval between the last 2 Gy FIR and the experiment.

### Assay for radiosensitivity

Cell survival after X-irradiation was measured by clonogenic assay. The cells (10^5^ cells per dish) were incubated at 37°C for 48 h in the growth medium and were irradiated at different doses ranging from 0 to 10 Gy. The cells were harvested with EDTA–trypsin immediately after irradiation. Subsequently, a constant number of cells were plated in each 60-mm tissue culture dish (Iwaki Glass, Chiba, Japan), and incubated at 37°C for 10–14 days (three plates in each radiation dose). After fixation with formalin and staining with 0.1% crystal violet, colonies consisting of 50 cells or more were counted under a light microscope, and the surviving fraction was determined.

All survival curves represent at least three independent experiments. The data were fitted to the single hit, multitarget formula, where survival (*S*) is related to dose (*D*) by the expression *S*=1−(1−e^−DD_0_^)^*n*^≒*n*e^−DD_0_^. *D*_0_ was used as a parameter to indicate the amount of irradiation required to reduce the survival fraction to approximately 0.37 from the survival curve.

### Flow cytometry

Cells were analysed during exponential growth. They were trypsinised, rinsed in PBS, and fixed in 70% ethanol. Before analysis, cells were spun out of ethanol and resuspended in 1.6 ml PBS, 0.2 ml propidium iodide (PI) 400 *μ*g ml^−1^ (Sigma Chemical Co., Poole, UK), and 0.2 ml RNase 1 mg ml^−1^ (Sigma). After incubation for 30 min at 37°C, the cells were spun down and resuspended in PBS. The cells were analysed by a FACScan flow cytometer (Becton Dickinson, San Jose, CA, USA) for DNA content using PI and a photomultiplier masked with a 610 nm long-pass filter. The data were analysed with a software program (Cell Quest; Becton Dickinson) with dead cells gated out using pulse processing. Two independent experiments were performed for each data set.

### RNA extraction

Total RNA was extracted from each cell line with the RNeasy Midi Kit (QIAGEN) according to the manufacturer's protocol. The extracted RNA was treated with RNase-Free DNase set (QIAGEN) to remove any contamination of genomic DNA according to the manufacture's protocol.

### Microarray design, production, and hybridisation

A 20 *μ*g portion of total RNA extracted from each radioresistant subline was labelled with Cy3, and RNA from each parent cell line was labelled with Cy5 as described elsewhere (Sakakura *et al*, 2002). We used the RIKEN human cDNA microarray, which contains 21168 genes. Hybridisation, washing, and scanning were carried out according to a published method (Sakakura *et al*, 2002). These slides were scanned on a ScanArray 5000 confocal laser scanner, and the images were analysed using IMAGENE (Bio Discovery, Los Angeles, CA, USA).

### Analysis of the data

To improve the accuracy of the data, we did the experiment twice, labelling the same RNA template in two separate reactions. Data were normalised to the reference standard by subtracting (in log space) the median observed value if other than zero. We used only data points that were reproducible. To this end, we developed a filtering program, PRIM (Kaota *et al*, 2001). After filtering was finished, we compared the results of the two experiments by calculating a Pearson's correlation coefficient. If the coefficient was equal to or greater than 0.7, we used the data in subsequent analysis. Ratio values from duplicate experiments were averaged, log-transformed (base 2), and stored in a table. Two-fold differences in up- or downregulated expression were used to identify altered genes. Hierarchical clustering and dendrogram figures were generated using Cluster and Tree-View software (http://rana.standford.edu).

### Quantitative RT–PCR

A quantitative RT–PCR assay was used to follow-up on microarray data. A 4 *μ*g portion of total RNA was used for creation of single-stranded cDNA using the SuperScript Preamplification System for First Strand cDNA Synthesis (Life Technologies Inc., Rockville, MD, USA). The cDNA was diluted and quantitatively equalised for PCR amplification. The TaqMan PCR method using a Gene Amp 5700 Sequence Detection System (Applied Biosystems, Foster City, CA, USA) was performed according to the manufacturer's instructions. We used the primers and probes provided as Assay-on-Demand Gene Expression Products (Applied Biosystems). We used beta-actin as an internal control gene to normalise the expression rate of each gene. The details have been described elsewhere (Sakakura *et al*, 2004). Each experiment was carried out three times and the average was calculated.

### Clinical specimens

Eight patients with advanced squamous cell carcinoma of the oesophagus (T3 or T4) treated with preoperative radiation therapy at Kyoto Prefectural University of Medicine were subjected to preliminary clinical analysis. Tissue samples were obtained from the tumour site for an initial diagnostic biopsy, and 10–40 *μ*g of total RNA was extracted, and cDNA was synthesised from 4*μ*g of total RNA as described. Preoperative irradiation was performed with a dose of 2 Gy per fraction to a total dose of 40 Gy. The effects of radiation therapy in the resected specimens were determined using the histopathologic criteria of the Japanese Society for Esophageal Disease. Grade 0 represents no radiotherapy effect; grade 1, slightly effective, with viable cells recognised as more than one-third cancer cells; grade 2, moderately effective, with less than one-third cancer cells; and grade 3, markedly effective, with no viable cancer cells. We compared the expression rates of the selected genes for group A (patients whose histological grade after radiotherapy was either 0 or 1) with those for group B (patients whose histological grade after radiotherapy was either 2 or 3). A statistical analysis was performed using the Mann–Whitney *U*-test. The results with *P*-values of less than 0.05 were considered to be statistically significant. All experiments with clinical samples were conducted under institutional guidelines from the Ministry of Health and Welfare.

## RESULTS

### Establishment of cell lines resistant to irradiation

The human oesophageal cancer cell lines were treated with FIR, and all cell lines survived. The cell populations surviving FIR were analysed for their radiosensitivity using a clonogenic assay to assess survival after a single dose of radiation. The survival curve parameters for all cell lines are shown in Table 1

**Table 1:** Clonogenic survival parameters fitting the data to a multitarget model

**Cell line**	***D*_0_**	**[95% confidence intervals]**	***n***	**[95% confidence intervals]**
TE-2				
Control	0.75	[0.68–0.84]	2.63	[1.49–4.63]
Resistant	1.05	[0.95–1.19]	2.85	[1.57–5.15]
TE-5				
Control	0.69	[0.57–0.85]	13.97	[4.05–48.24]
Resistant	0.73	[0.65–0.85]	9.25	[4.21–20.29]
TE-9				
Control	1.14	[1.00–1.32]	1.76	[1.21–2.55]
Resistant	1.71	[1.53–1.93]	1.60	[1.15–2.23]
TE-13				
Control	1.49	[1.39–1.61]	2.42	[1.76–3.34]
Resistant	1.83	[1.70–1.98]	2.61	[1.97–3.45]
KYSE170				
Control	1.31	[1.17–1.49]	2.46	[1.55–3.90]
Resistant	1.76	[1.49–2.17]	2.73	[1.38–5.37]
KYSE180				
Control	0.91	[0.84–0.99]	2.43	[1.74–3.38]
Resistant	0.97	[0.89–1.07]	2.02	[1.43–2.84]

*D*_0_ was used as a parameter to indicate the amount of irradiation required to reduce the survival fraction to approximately 0.37 from the survival curve.

. There was a significant increase in radioresistance in the TE-2R, TE-9R, TE-13R, and KYSE-170R sublines compared to parental cell lines. Therefore, we considered these four sublines (TE-2R, TE-9R, TE-13R, and KYSE-170R) as radioresistant. All radioresistant sublines maintained a radioresistant phenotype for at lease 3 months after the cessation of FIR (data not shown).

### Cell cycle analysis

Cells in S phase tend to be more radiosensitive than cells in G1, and cells in G2–M are the most sensitive of all (Sinclair and Morton, 1966). Therefore, we examined the cell cycle distribution of cell lines using flow cytometry. The results in Table 2

**Table 2:** Cell cycle distributions of the cell lines

**Cell line**	**G1**	**S**	**G2/M**
TE-2			
Control	54.9	18.1	27
Resistant	49	22.5	28.5
TE-9			
Control	57.3	16.1	26.6
Resistant	60.8	17.2	21.9
TE-13			
Control	49.5	23.9	26.6
Resistant	53.1	24.7	22.2
KYSE-170			
Control	51.8	17	31.2
Resistant	56	16.7	27.3

show that, when in exponential growth, the lines have very similar cell cycle distributions and thus cannot be the explanation for the altered radiosensitivity of resistant cells. Other experiments also showed similar results (data not shown).

### Gene selection from cDNA microarray data analysis

We performed global expression analysis of 21168 genes using a high-density cDNA microarray and compared the four profiles of parent lines and radioresistant sublines. We identified 47 genes as differentially expressed in at least three of the four radioresistant sublines. In all, 19 genes were upregulated (Table 3

**Table 3:** Upregulated genes in radioresistant sublines in comparison to parent cell lines

**Function**	**Accession**	**Description**	**TE-2**	**TE-9**	**TE-13**	**KYSE170**
Apoptosis, inflammatory response	AA418021	ICEBERG caspase-1 inhibitor (ICEBERG)	2.18	1.04	4.18	2.06
Apoptosis	R44417	baculoviral IAP repeat-containing 2 (BIRC2)	2.23	2.28	1.57	2.96
Blood coagulation, cell growth	AA284669	plasminogen activator, urokinase (PLAU)	2.64	2.48	1.11	3.23
Carbohydrate metabolism	AA775378	mannosyl (alpha-1,3-)-glycoprotein beta-1,2-*N*-acetylglucosaminyltransferase (MGAT1)	2.06	8.18	0.45	3.06
Cell adhesion, immune response	AA458965	natural killer cell transcript 4 (NK4)	7.03	8.53	1.03	2.28
DNA metabolism	R60343	5′ nucleotidase (CD73) (NT5)	2.21	0.54	4.45	4.26
Electron transport	AA418907	cytochrome P450, subfamily I (aromatic compound-inducible), polypeptide 1 (CYP1A1)	7.19	2.19	0.41	2.67
Electron transport	R33030	glucose regulated protein, 58 kDa (GRP58)	2.80	2.11	1.51	3.22
Electron transport	AA430497	protein disulphide isomerase related protein (calcium-binding protein, intestinal-related) (ERP70)	9.78	2.92	1.11	2.94
Inflammatory response	R80217	prostaglandin-endoperoxide synthase 2 (prostaglandin G/H synthase and cyclooxygenase) (PTGS2) (COX-2)	2.16	3.17	4.16	1.29
Protein amino-acid phosphorylation	AA447574	protein kinase C, zeta (PRKCZ)	2.00	10.04	0.92	3.70
Protein amino-acid phosphorylation	N94921	receptor tyrosine kinase-like orphan receptor 2 (ROR2)	2.05	0.95	5.06	2.30
Protein amino-acid phosphorylation	H74007	serum/glucocorticoid regulated kinase (SGK)	4.04	2.20	2.51	1.00
Regulation of transcription, DNA-dependent	AA293192	putative nucleic acid binding protein RY-1 (RY1)	2.17	2.32	1.00	2.37
Transport	AA400973	lipocalin 2 (oncogene 24p3) (LCN2)	3.01	2.33	1.31	5.47
tRNA processing	AA156571	alanyl-tRNA synthetase (AARS)	3.81	2.29	0.66	2.39
Unclassified	T67069	GTP binding protein 2 (GTPBP2)	2.77	2.42	1.10	2.96
Unclassified	N90081	tripartite motif protein TRIM8 (TRIM8)	2.28	2.35	0.99	6.68
Unclassified	AA453410	tumour necrosis factor receptor superfamily, member 10b (TNFRSF10B)	2.08	19.12	0.92	3.03

Representative functions of the genes as previously reported, accession number, gene name, and fold change of each radioresistant subline compared to their parent cell line are shown. Only the genes that showed more than two-fold change in at least three of the four radioresistant sublines are sorted by cDNA microarray data.

), and 28 were downregulated (Table 4

**Table 4:** Downregulated genes in radioresistant sublines in comparison to parent cell lines

**Function**	**Accession**	**Description**	**TE-2**	**TE-9**	**TE-13**	**KYSE170**
Apoptosis	W45688	caspase 6, apoptosis-related cysteine protease (CASP6)	0.09	0.14	0.04	0.04
Carbohydrate metabolism	AA193116	glycerol-3-phosphate dehydrogenase 1 (soluble) (GPD1)	1.64	0.37	0.44	0.37
Cell adhesion	H97778	cadherin 1, type 1, E-cadherin (epithelial) (CDH1)	0.05	0.25	0.07	0.02
Cell adhesion	AA425217	cadherin 3, type 1, P-cadherin (placental) (CDH3)	0.05	0.22	0.03	0.02
Cell adhesion	N63057	protocadherin 9 (PCDH9)	0.43	0.07	0.69	0.36
Cell growth, cell–cell signalling	W56771	endometrial bleeding associated factor (left–right determination, factor A; transforming growth factor beta superfamily) (EBAF)	0.45	1.00	0.19	0.40
Epidermal differentiation	W23757	keratin 13 (KRT13)	0.37	0.30	3.32	0.19
Intracellular protein transport	R28020	RAB2, member RAS oncogene family (RAB2)	0.07	0.20	0.03	0.03
Intracellular signalling cascade	R91570	signal transducer and activator of transcription 4 (STAT4)	0.19	0.12	0.09	0.06
Ion transport	T52201	chloride intracellular channel 2 (CLIC2)	0.60	0.40	0.48	0.32
Metabolism	AA668821	chitinase 3-like 2 (CHI3L2)	0.42	0.45	0.61	0.15
Mitosis, regulation of CDK activity	AA608568	cyclin A2 (CCNA2)	0.36	0.42	0.44	2.90
Neurogenesis	AA425008	cerebellin 1 precursor (CBLN1)	1.09	0.37	0.20	0.21
Oncogenesis	W69960	Wolf–Hirschhorn syndrome candidate 1 (WHSC1)	0.12	0.10	0.05	0.10
Protein amino-acid phosphorylation	AA598841	natriuretic peptide receptor A/guanylate cyclase A (atrionatriuretic peptide receptor A) (NPR1)	0.46	0.34	0.74	0.41
Proteolysis and peptidolysis	H94487	cathepsin E (CTSE)	0.79	0.39	0.34	0.12
Regulation of cell cycle	H73724	cyclin-dependent kinase 6 (CDK6)	0.27	0.53	0.15	0.10
Regulation of transcription, DNA dependent	H82435	myeloid/lymphoid or mixed-lineage leukaemia 3 (MLL3)	0.29	0.46	0.39	0.56
Regulation of transcription, DNA dependent	AA480906	protein kinase C binding protein 1 (PRKCBP1)	0.40	0.23	1.10	0.48
Signal transduction	H93332	apolipoprotein B (including Ag(x) antigen) (APOB)	0.37	0.49	0.56	0.46
Skeketal development	W58032	frizzled-related protein (FRZB)	0.67	0.45	0.43	0.35
Steroid metabolism	N92404	sulphotransferase, oestrogen-preferring (STE)	1.00	0.28	0.18	0.30
Unclassified	AA454819	mitogen-activated protein kinase 3 (MAPK3)	0.21	0.29	0.07	0.11
Unclassified	AA450024	T-cell lymphoma invasion and metastasis 2 (TIAM2)	0.16	0.17	0.04	0.07
Unclassified	R89492	cytochrome P450, subfamily IIC (mephenytoin 4-hydroxylase), polypeptide 9 (CYP2C9)	0.49	0.50	0.55	0.29
Unclassified	AA487505	immunoglobulin superfamily, member 4 (IGSF4)	0.39	0.28	0.55	0.44
Unclassified	AA490903	pleckstrin homology, Sec7 and coiled/coil domains, binding protein (PSCDBP)	0.43	0.40	0.83	0.49
Unclassified	AA417616	B-cell CLL/lymphoma 7A (BCL7A)	0.35	0.22	0.13	0.09

Representative functions of the genes as previously reported, accession number, gene name, and fold change of each radioresistant subline compared to their parent cell line are shown. Only the genes that showed more than two-fold change in at least three of the four radioresistant sublines are sorted by cDNA microarray data.

). Upregulated genes were associated with apoptosis and inflammatory response (ICEBERG, BIRC2, and COX-2), DNA metabolism (CD73), and cell growth (PLAU). Other areas of upregulation included electron transport (CYP1A1, GRP58, and ERP70) and protein amino-acid phosphorylation (PRKCZ, ROR2, and SGK). Downregulated genes were associated with apoptosis (CASP6), cell adhesion (CDH1, CDH3, and PCDH9), and transcription (MLL3 and PRKCBP1), as well as cell cycle (CDK6 and CCNA2).

**Figure 1:**
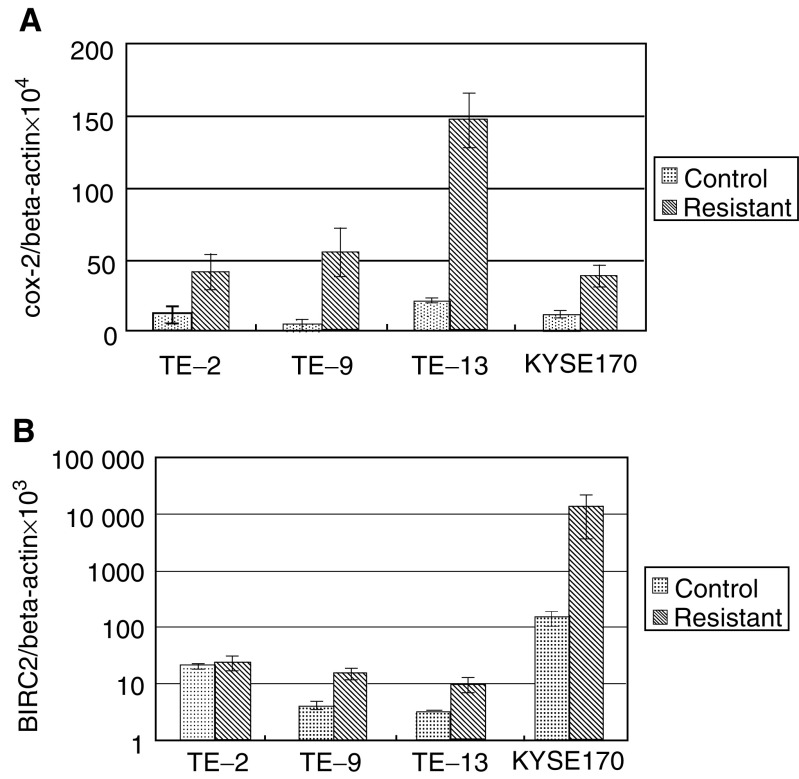
Quantitative RT–PCR analysis of the genes selected for cDNA microarray data validation. (**A**) Relative expression rate of COX-2 was normalised to beta-actin. (**B**) Relative expression rate of BIRC2 was normalised to beta-actin. Data represent mean±standard deviation (*n*=3).

To verify the expression of genes identified in microarray experiments, real-time quantitative RT–PCR was performed using the same RNA as that used in the microarray analysis. We found that the results were in good agreement with those from the microarray data. Typical data for COX-2 and BIRC2 are shown in Figure 1

.

### Effects of radiotherapy and expression of selected genes in clinical specimens

**Figure 2:**
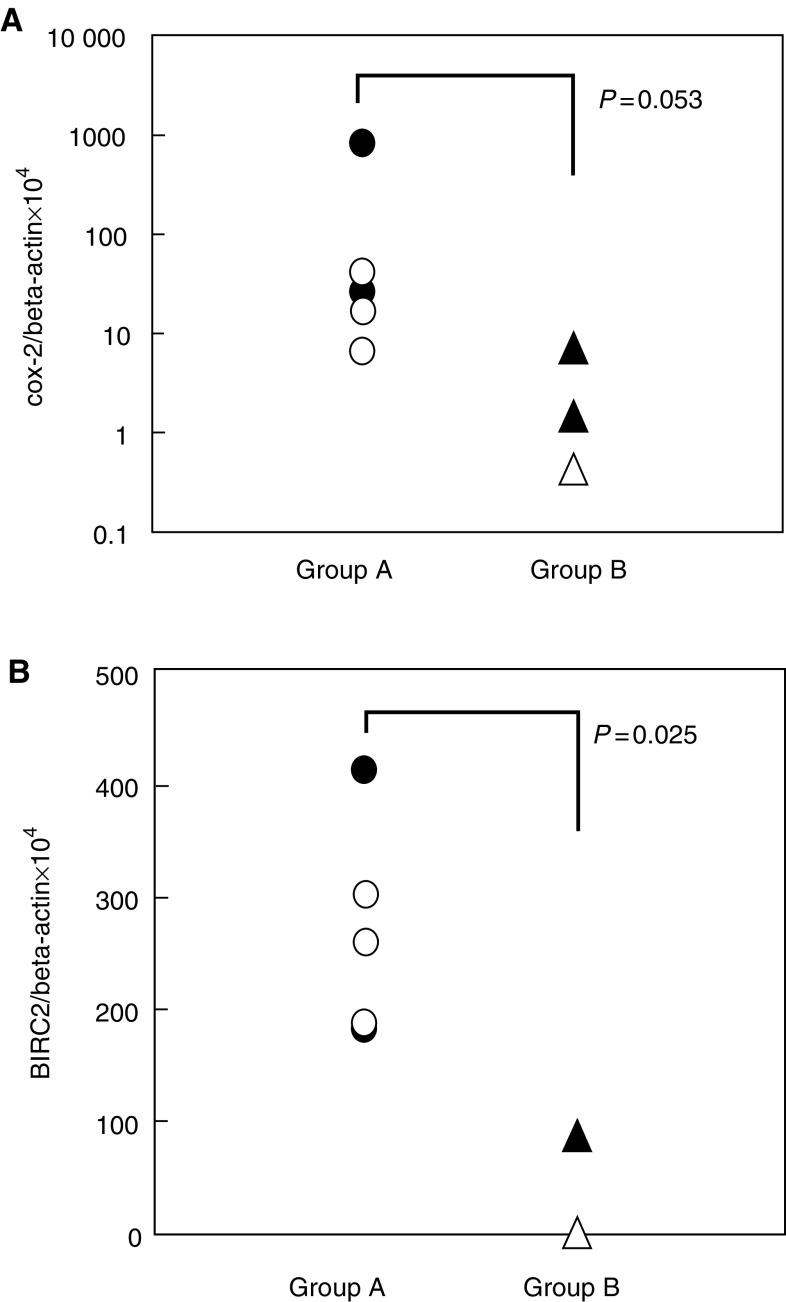
Effect of radiotherapy and expression rate of COX-2 (**A**) and BIRC2 (**B**) in clinical specimens. Expression rates were normalised to beta-actin. The grade of radiotherapy effect was determined based on the histopathological criteria of the Japanese Society for Esophageal Diseases. Grade 0 (•), ineffective; Grade 1 (○), slightly effective; Grade 2 (▴), moderately effective; and Grade 3 (▵), markedly effective. Group A included patients whose histological grade after radiotherapy was either 0 or 1, and group B included patients whose grade was either 2 or 3. The statistical analysis was performed using the Mann–Whitney *U*-test.

In eight cases of squamous cell carcinoma of the oesophagus, COX-2 and BIRC2 mRNA expressions of pretreatment biopsy samples were assayed by quantitative RT–PCR. Although the expression rates of COX-2 for group A were not significantly higher than those for group B (Figure 2A

, *P*=0.053), the expression rates of BIRC2 for group A were significantly higher than those for group B (Figure 2B, *P*=0.025). These results suggest that genes, which are overexpressed in radioresistant sublines, may reflect clinical specimens of radioresistant cases.

## DISCUSSION

Squamous cell oesophageal cancer shows intermediate-grade radiosensitivity, but even within the same histologic type, there is heterogeneous radiosensitivity. Radioresistance is an obstacle in cancer therapy and affects the curability of patients. Therefore, it is necessary to elucidate the radioresistance mechanisms to improve prognosis. Some investigators have tried to identify a set of genes related to radiosensitivity by comparing expression profiles of radiosensitive and radioresistant tumours obtained as clinical samples before treatment using a cDNA microarray (Hanna *et al*, 2001; Kitahara *et al*, 2002). However, it is very difficult to distinguish whether the radioresistance was inherent in the patient or the tissue itself or if it was acquired after cancer therapy. Thus, we thought that it is advantageous to compare cells of the same origin with and without radioresistance.

Radioresistant sublines were obtained by exposing the parent lines to repeated fractions of 2 Gy, with 5–7 days recovery allowed between each fraction. This resulted in a statistically significant decrease in the radiosensitivity of the four exposed sublines as measured by clonogenic assay (Table 1). One explanation for the increased radioresistance might be an adaptive response to radiation, which has been observed in human lymphocytes (Olivieri *et al*, 1984). However, we observed that the radioresistant sublines maintained a radioresistant phenotype for at least 3 months after cessation of FIR. Russell *et al* (1995) explained that the selective pressure of repeated irradiation favours the emergence of radioresistant clones. Comparing the radioresistant sublines with their parent cell lines, we could truly examine genes associated with radioresistance.

A total of 19 upregulated (Table 3) and 28 downregulated (Table 4) genes were common to radioresistant sublines. With regard to radioresistance, malignant characteristics of cancer cells such as resistance to apoptosis, reduced intracellular adhesion, increased cell–matrix adhesion, and modified cell cycle are considered to be important features. Several of the 47 genes have been previously reported to be related to these features. The possible involvement of other genes listed in Tables 3 and 4 to radioresistance has not yet been reported. Other sequence tags (ESTs) can be expected to be involved in radioresistance, but the precise function of each gene remains unclear, so further study would be necessary to clarify this.

### Genes related to apoptosis and cell cycle

Prostaglandin-endoperoxide synthase (PTSG), also know as cyclooxygenase (COX), is the key enzyme in prostaglandin biosynthesis, and acts both as a dioxygenase and as a peroxidase. There are two isozymes of COX: a constitutive COX-1 and an inducible COX-2, which differ in their regulation of expression and tissue distribution. COX-2 is expressed in a limited number of cell types and regulated by specific stimulatory events, suggesting that it is responsible for the prostanoid biosynthesis involved in inflammation and mitogenesis. Recent studies have highlighted the relevance of COX-2 in human carcinogenesis (Eberhart *et al*, 1994; Ristimaki *et al*, 1997; Hida *et al*, 1998; Koga *et al*, 1999; Tucker *et al*, 1999). In oesophageal cancer, increased levels of COX-2 have also been reported (Hwang *et al*, 1998; Wilson *et al*, 1998; Katja *et al*, 1999; Shamma *et al*, 2000). Although the role of COX-2 in oesophageal cancer is not clear, it is possible that COX-2 is involved in the regulation of cell survival and maintenance of growth because COX-2 inhibitors are known to induce apoptosis and inhibit growth in oesophageal carcinoma cells (Katja *et al*, 1999). Furthermore, Pyo *et al* (2001) showed that selective COX-2 inhibitors enhanced the radiosensitivity of rat intestinal epithelial cells, which were stably transfected with COX-2 cDNA, and Nakata *et al* (2004) reported similar findings with human A431 epidermoid carcinoma. Our data suggest that COX-2 may play an important role in radioresistance induced by FIR, and COX-2 inhibitors may prevent tumour cells from acquiring radioresistance. Furthermore, in our preliminary clinical study, the expression rate of COX-2 tended to reflect the effect of radiotherapy (Figure 2A, *P*=0.053). Additional investigation will be necessary to confirm this hypothesis.

BIRC2, also known as cIAP1, is a gene that encodes an antiapoptotic molecule in the IAP family: oesophageal squamous cell carcinoma cell lines overexpressing this gene were resistant to apoptosis induced by chemotherapeutic reagents (Imoto *et al*, 2001). Furthermore, Imoto *et al* (2002) reported that the expression of cIAP1 was correlated with resistance of cervical cancers to radiotherapy. In our study, the expression rates of BIRC2 were significantly higher in patients whose histological grade after radiotherapy was either 0 or 1 than those patients whose histological grade was either 2 or 3 (Figure 2B, *P*=0.025). These data also suggest that we may predict the effect of radiotherapy using the expression rate of BIRC2.

Upregulation of ICEBERG, a caspase-1 inhibitor, and downregulation of caspase 6 (CASP6) are extremely intriguing. ICEBERG proteins are intracellular regulators of caspase-1 activation and could play a role in the regulation of IL-1*β* secretion and NF-*κ*B activation during the proinflammatory cytokine response (Humke *et al*, 2000; Druilhe *et al*, 2001), NF-*κ*B is a well-defined radiation-responsive transcription factor (Thanos and Maniatis, 1995). Furthermore, NF-*κ*B is associated with the cell cycle, and we found that cyclin A2 (CCNA2) was downregulated. However, genes such as p53, cyclin D1, and NF-*κ*B did not differ in our study, and the cell cycles of radioresistant sublines were similar to that of parent cell lines (Table 2). Another mechanism of cell cycle regulation may exist.

### Genes related to growth and metabolism

CD73, also termed 5′-nucleotidase, is a signalling pathway metabolite in the immune response of lymphocytes. This ectoenzyme catalyses the conversion of purine and pyrimidine ribo- and deoxyribonucleoside monophosphates (AMP, GMP, IMP) and leads to an elevation of the corresponding nucleosides (adenosine) in the extracellular space, perhaps modulating neuronal signalling and vascular perfusion, CD73 has also been called a cellular motility factor. There is an increasing amount of evidence for a modulatory role of PKC-mediated CD73 activity in ischaemia, regeneration and repair, glioma cell proliferation, and tissue invasion. Ludwig *et al* (1999) reported that CD73 expression was correlated with expression of protein kinase C (PKC) and epidermal growth factor receptor (EGFR). Protein kinase C is a known tumour metabolite in several proliferation-promoting pathways of EGF receptor signalling. Furthermore, Galmarini *et al* (2002) reported that CD73-positive acute myeloid leukaemia (AML) patients had worse disease-free survival and overall survival than negative patients, and suggested that CD73 expression might be a mechanism of resistance to cytarabine. To our knowledge, there have been no reports of an association between radioresistance and CD73.

### Genes related to cell adhesion and motility

In our study, E-cadherin (CDH1), P-cadherin (CDH3), and protocadherin 9 (PCDH9) were downregulated in radioresistant sublines. However, Akimoto *et al* (1998) reported that E-cadherin expression was increased significantly in human squamous cell carcinoma after irradiation. This difference might be due to fractionated irradiation and acquisition of radioresistance. Some studies have shown that reduced E-cadherin expression was correlated with a high incidence of cancer metastasis and invasion (Doki *et al*, 1993; Kadowaki *et al*, 1994), and radiation enhanced the ability to form metastases (Sheldon and Fowler, 1976). Our results also suggest that the tumour cells that acquired radioresistance may have higher invasive ability and metastatic potential. Recently, an association between E-cadherin and COX-2 has been reported (Tsujii and Dubois, 1995), and Noda *et al* (2002) found that COX-2 inhibitors affect the transcription of E-cadherin.

Gene expression changes related to apoptosis, cell cycle, growth metabolism, and cell adhesion are likely to contribute to the complexity of radioresistance. It is unlikely that this phenomenon can be explained by altered expression of a single gene: whether this combination of gene expression changes leads to radioresistance of oesophageal cancers, however, is a matter to be investigated further. Identification of such genes could lead to new therapeutic targets.
